# Oral Session 03: CNS Risk

**DOI:** 10.1093/jrr/rrt207

**Published:** 2014-03

**Authors:** Livio Narici, Gregory A. Nelson

**Affiliations:** 1 Department of Physics, University of Rome Tor Vergata and INFN Tor Vergata, Via della Ricerca Scientifica 1, 00133 Rome, Italy; 2 Loma Linda University, 11175 Campus St., Loma Linda, CA 92354, USA

**Keywords:** CNS risk, cosmic radiation, hippocampal function, mixed beam irradiation, oxidative stress

## Abstract

Exposure to space radiation may have impacts on brain function, either during or following missions. It is most important to determine how low doses of protons and high-LET irradiation elicit changes in brain function. Within this framework, the role of oxidative stress should also be assessed, as well as other possible interaction mechanisms involving, e.g., genetic, environmental, and sex-dependent risk factors. The hippocampus is particularly susceptible to radiation. It plays an essential role in memory formation and consolidation and is one of the most investigated brain components for its responses to radiation. The hippocampus is also one of the first brain structures to be damaged in the pathogenesis of Alzheimer's disease, an important potential late impairment following irradiation. In ‘Section 3: CNS risk’, six papers have been presented focused on these issues. For details the reader is directed to the specific papers. Here a very short summary follows.

## SHORT SUMMARY

Experiments have been carried out *in vitro* and/or *in vivo*, and in both cases dose has been revealed as playing an important role. Mice and rats are usually used as models, but caution should be used in extrapolating these results to the human health effects associated with ionizing radiation exposure, a connection that still needs further investigation [1].

When doses exceed mission-relevant doses (≥0.5 Gy) indications of cognitive deficits become obvious. HZE irradiation inhibits overall neurogenesis at high doses (>1 Gy) but at lower doses irradiation alters the lineage and phenotypes of neural precursor cells [2]. Also, after high doses (0.5 Gy, 2 Gy) there appears to be increases in Alzheimer-like deficits in Alzheimer's disease (AD) mouse models [3].

At lower doses a more complex picture appears. Genetic and age effects, and dependence on time-points and also on the radiation history (mixed fields) show up. There are, e.g., unique proteins expressed in irradiated rats that have either good or poor spatial memory performance [4]. While total-body mouse irradiation elicits early or late alterations in a few AD-related genes, it appears not to have significant impacts on spatial learning and memory or on AD-like pathological changes [1]. Novel object recognition is impaired with an age-dependent dose–response curve. There are suggestions that genetic factors might modulate the direction of the effects of space irradiation on hippocampal function. Behavioral training may modify electrophysiological endpoints. Radiation effects on cognition are not necessarily detrimental at all time-points or in all cognitive tests [5]. Microenviron-mental changes, including innate immune system features, appear to interact with neurogenesis [2].

Mixed-beam irradiation is shown to increase the complexity of responses. Time between exposures, as well as radiation type, play important roles. In some cases an adaptive response after mixed-beam irradiation appears to lead to a ‘protective’ effect [2]. Low-LET and high-LET radiation differentially affect neural precursor cell lineages [2].

Electrophysiology on isolated hippocampal slices using patch clamp and field-recording techniques show that radiation effects on synaptic excitability and plasticity in the hippocampus differ between distinct fields and neuronal populations (CA1 vs DG). Overexpression of mitochondrial catalase (MCAT) suppresses the effects of radiation. Long-term potentiation (tissue level memory model) is affected differently by ^1^H, ^28^Si and ^56^Fe ions. In studies of oxidative stress it has been shown *in vitro* that neural stem cells show marked radiation-induced ROS/RNS responses that are persistent for weeks, and that this is LET dependent. *In vivo* results indicate that irradiation initiates a strong antioxidation response at 2 weeks, which returns to baseline after 4 weeks. Finally, the microanatomy of neurons appears to be altered after irradiation, resulting in less dendritic complexity and fewer dendritic spines.

Overall, the presented studies clearly document that low (mission-relevant) doses of charged particles induce:

- alterations in neurogenesis and functional integration of new neurons;
- persistent oxidative stress;
- reduced neuronal dendrite complexity and spine density;
- altered synaptic plasticity in region- and ion-specific patterns;
- altered neuron intrinsic properties;
- degradation of spatial memory performance;
- alteration in several AD-related genes;
- impaired hippocampus-dependent behavior;
- increased activation of microglia and altered chemokines;
- inappropriate signal processing after radiation (suggesting that compensatory responses may be elicited in the brain).

High-fidelity hippocampus models have finally been proposed as a tool to predict some responses using measured parameters [6].
